# Determining the Sub-Cellular Localization of Proteins within *Caenorhabditis elegans* Body Wall Muscle

**DOI:** 10.1371/journal.pone.0019937

**Published:** 2011-05-17

**Authors:** Barbara Meissner, Teresa Rogalski, Ryan Viveiros, Adam Warner, Lorena Plastino, Adam Lorch, Laure Granger, Laurent Segalat, Donald G. Moerman

**Affiliations:** 1 Department of Zoology, University of British Columbia, Vancouver, British Columbia, Canada; 2 Michael Smith Laboratories, University of British Columbia, Vancouver, British Columbia, Canada; 3 CNRS-CGMC, Universite Lyon-1 Claude Bernard, Villeurbanne, France; University Medical Center Groningen, The Netherlands

## Abstract

Determining the sub-cellular localization of a protein within a cell is often an essential step towards understanding its function. In *Caenorhabditis elegans*, the relatively large size of the body wall muscle cells and the exquisite organization of their sarcomeres offer an opportunity to identify the precise position of proteins within cell substructures. Our goal in this study is to generate a comprehensive “localizome” for *C. elegans* body wall muscle by GFP-tagging proteins expressed in muscle and determining their location within the cell. For this project, we focused on proteins that we know are expressed in muscle and are orthologs or at least homologs of human proteins. To date we have analyzed the expression of about 227 GFP-tagged proteins that show localized expression in the body wall muscle of this nematode (e.g. dense bodies, M-lines, myofilaments, mitochondria, cell membrane, nucleus or nucleolus). For most proteins analyzed in this study no prior data on sub-cellular localization was available. In addition to discrete sub-cellular localization we observe overlapping patterns of localization including the presence of a protein in the dense body and the nucleus, or the dense body and the M-lines. In total we discern more than 14 sub-cellular localization patterns within nematode body wall muscle. The localization of this large set of proteins within a muscle cell will serve as an invaluable resource in our investigation of muscle sarcomere assembly and function.

## Introduction

Mutations in sarcomeric proteins are implicated in at least 20 different skeletal muscle diseases in humans [1]. Unfortunately, the pathophysiology for most of these diseases is poorly understood due, in part, to our lack of knowledge about the normal process of myofilament assembly or stability. The building of a sarcomere is a dynamic, multifaceted and precisely regulated process and working with model systems gives us the opportunity to study the assembly of this evolutionarily conserved structure in detail [2]–[8]. The free-living nematode *Caenorhabditis elegans* has proven to be an exceptionally good model system to study the development of muscle [8]–[13]. Work in several laboratories including ours has focused on sarcomere assembly in body wall muscle, specifically on the early events that occur at the muscle cell membrane (reviewed in [8]). The aim of these studies is to describe as fully as possible a parts list for the assembly and organization of myofilaments within the body wall muscle of *C. elegans*.

In *C. elegans* dense bodies and M-lines attach to actin filaments and myosin filaments, respectively, and are homologs of the Z-lines and M-lines in vertebrate striated muscle [14]. In adult muscle cells both dense bodies and M-lines are finger-like projections that extend from the muscle cell membrane into the cytoplasm. Dense bodies are analogous to vertebrate integrin-mediated attachments between the ECM and the actin cytoskeleton. They are composed of cytoskeletal adaptor proteins including vinculin, alpha-actinin, talin, PINCH, Kindlin, ILK, and actopaxin/alpha-parvin which link the cytoplasmic domain of integrin and the actin filaments in the myofilament lattice [14]–[20]. The M-lines contain many of the same membrane-proximal adaptors, but lack vinculin. The membrane-distal region of the M-line lacks the dense body protein alpha-actinin, but does include the M-line specific protein UNC-89 [21]. Given their protein composition and functions, dense bodies and M-lines are both analogous and homologous to vertebrate integrin mediated adhesion plaques, commonly called focal adhesions (FA's) in tissue culture cells [22], [23].

The regulatory steps that coordinate the assembly of adhesion plaques into functional attachment structures capable of enduring and transmitting mechanical stress are largely undefined. In *C. elegans* genetic screens for Pat (Paralyzed, Arrested elongation at Two fold) mutants has identified animals with defects in sarcomere assembly, and these have proven a powerful aid in identifying novel focal adhesion proteins and for the investigation of their functions *in vivo* (for example, see [18]–[20], [24]). Wild type embryos proceed through a series of elongations while still in the egg and do not hatch until they are three fold in length. Mutants with early, severe defects in sarcomere assembly fail to begin normal embryonic movements at mid-embryogenesis, and soon after this point the elongation process stops prematurely at the two fold length.

Necessary prerequisites for understanding sarcomere assembly include the identification of all proteins within muscle and more specifically the identification and localization of all proteins associated with the sarcomere. Using a combination of Serial Analysis of Gene Expression (SAGE) and Affymetrix GeneChip data we have determined that developing body wall muscle cells express a minimum of 4,430 genes (excluding ribosomal genes) [25], [26]. The samples used for these studies were purified muscle cells from late developing embryos at a time when sarcomeres are just being formed (a two hour window). The list of 4,430 genes is based on observations from two SAGE libraries and three Affymetrix GeneChip libraries and only includes genes observed with both platforms and in at least three separate libraries (http://tock.bcgsc.bc.ca/cgi-bin/sage160). Known sarcomere components are well represented among the 4,430 genes expressed in muscle. The aim of the current study was to begin to determine which of the many novel proteins expressed in muscle are located within sarcomeres.

Since the first publication by Chalfie et al [27] using GFP to monitor gene expression in the touch cells of *C. elegans*, GFP-tagging has become a standard tool in the arsenal of cell biologists. Studies in yeast were the first to use this method to analyze protein localization on a global scale [28]. Our goal here was to carry out an analogous study, albeit on a smaller scale, within *C. elegans* muscle. Our strategy was to use the Gateway cloning system [29], [30] and the commercially available ORFeome library [31], [32] to construct clones expressing GFP-tagged proteins under the control of a muscle specific promoter. These constructs were then injected into animals and their expression pattern and localization within body wall muscle monitored.

Knowing the precise location of a particular novel protein within a cell can lead to a better understanding of its function, or at the very least, lead to suggested experiments to test function. In *C. elegans* the exquisitely organized sarcomeres within the body wall muscle cells offer an opportunity to identify the precise position of proteins within these substructures. In this study we have determined the sub-cellular localization of 227 GFP-tagged proteins in body wall muscle cells. Most of these proteins have extensive sequence similarity to, or are, clear orthologs of human proteins. At least 80 of these proteins are newly identified components of the muscle cell sarcomere. This more than doubles the number of proteins previously shown to localize to these structures and identifies many genes for further analysis.

## Results

### Construction and analysis of GFP-tagged ORF clones using Gateway technology

In this study we have determined the sub-cellular localization of 227 proteins in the body wall muscle cells of *C. elegans*. The genes we targeted were chosen from several sources including our RNAi screen for myofilament disorganization [26] published muscle expressome data [25] and promoter studies [33]. In addition we analyzed the data from several new SAGE libraries to identify genes with at least 3 fold enriched-expression in embryonic muscle cell libraries compared to whole embryo libraries (data available at http://elegans.bcgsc.bc.ca). We mainly chose proteins with human homologs and where there was no described function. Also, we tried to eliminate proteins with previously determined sub-cellular distribution. The final criterion was that the coding sequence had to be present in the ORFeome library utilized to generate the GFP-tagged proteins [32]. The genes chosen for analysis are listed in Table S1.

**Figure 1:**
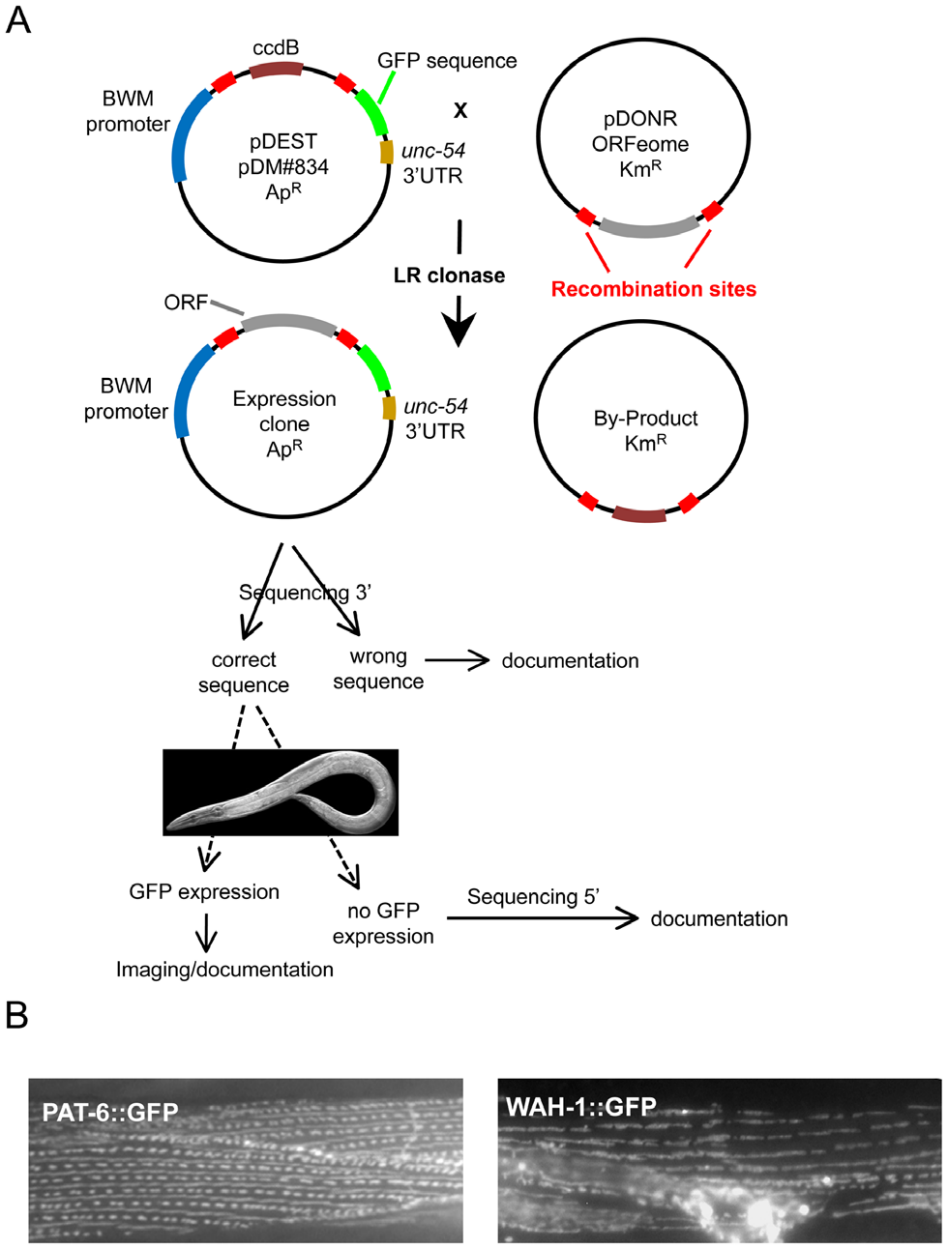
A schematic diagram describing the Gateway cloning protocol used to generate expression clones. The Gateway cloning protocol used in this study is outlined in (A). The sub-cellular localization observed for the PAT-6::GFP and WAH-1::GFP expressing Gateway clones are shown in (B).

**Figure 2:**
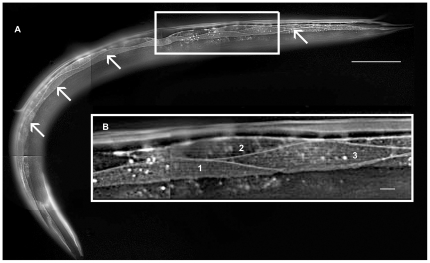
GFP-tagged protein expression in the body wall muscle of an adult *C. elegans* hermaphrodite. GFP-tagged protein expression in one body wall muscle quadrant in an adult hermaphrodite from strain VH25 is shown in panel A. The other 3 quadrants are out of the plane of focus. The anterior (head) of the animals is in the lower left-hand corner and the posterior (tail) of the animal is at the top right. The arrows point to the two rows of body wall muscle cells that form the muscle quadrant. Panel B is a higher magnification of the body wall muscle quadrant showing three symmetrical body wall muscle cells labeled 1, 2 and 3. The GFP-tagged protein in this strain (a gift from H. Hutter) localizes to the muscle cell membrane. The bar in panel A represents 100 µm and the bar in panel B represents 10 µm.

Fluorescent GFP-tagged proteins are commonly used in protein localization studies. In this study we expressed GFP-tagged proteins under the control of a muscle cell specific promoter and determined their sub-cellular localization using fluorescence microscopy. The *C. elegans* ORFeome collection contains about 12,500 protein-encoding open reading frames available as Gateway Entry clones [32], which represents about 60% of the *C. elegans* annotated ORF's. This valuable resource allowed us to utilize the Gateway recombination cloning system to obtain our GFP fusion proteins. To do this we constructed our own custom-made destination vector to express target genes exclusively in *C. elegans* muscle cells (described in Materials and Methods). The basic project workflow is outlined in Figure 1A. Briefly, the protein coding sequence (ORF) from the donor clone was inserted between the muscle promoter and the GFP coding sequence in the destination clone. All expression clones generated via the LR Gateway reaction were sequenced across the ORF/GFP junction to confirm that the introduced coding sequence was in frame with the GFP coding sequence. A total of 83 clones did not pass our initial sequencing check and were therefore excluded from further analysis. Transgenic animals expressing GFP fusion proteins were generated by microinjection [34] and GFP expression in these animals was analyzed *in vivo*. Figure 2A shows an example of a GFP-tagged protein being expressed in the body wall muscle cells of an adult hermaphrodite. One muscle quadrant consisting of two rows of spindle shaped muscle cells can be seen in the plane of focus. Several muscle cells from this quadrant are shown in greater detail in Figure 2B.

**Figure 3:**
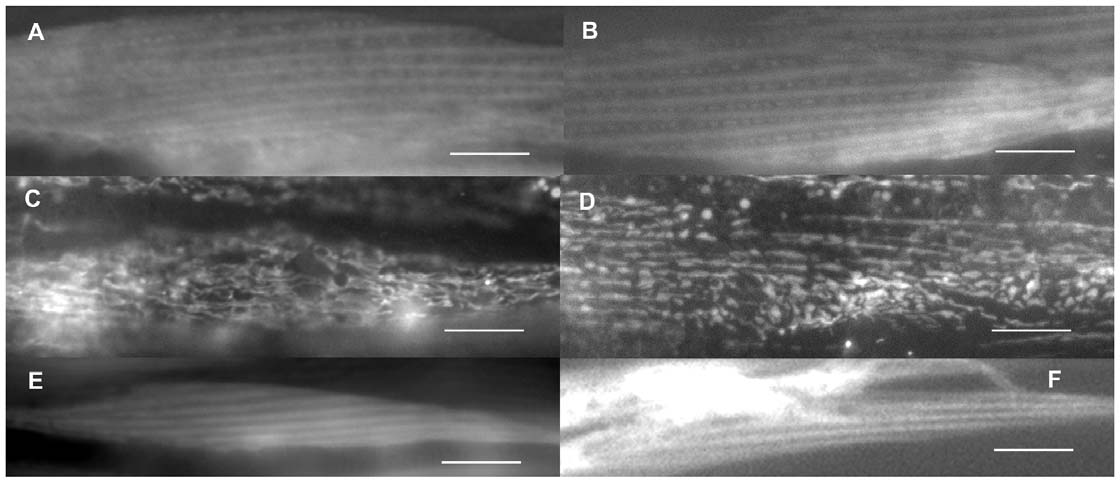
Comparison of GFP localization of proteins expressed from Gateway versus genomic clones. The sub-cellular localization for the T05D4.1::GFP (A, B), D2030.5::GFP (C, D) and F28H1.2::GFP (E, F) proteins expressed from gateway clones using a muscle-specific promoter (A, B and C) or from genomic clones using endogenous promoters (B, D and F). Each panel shows a single symmetrical, spindle–shaped body wall muscle cells exhibiting GFP-tagged protein localization. Bars in A and B represent 10 µm. Bars in C through F represent 20 µm.

To validate the applicability of this approach we tested two proteins with known expression patterns. The PAT-6 protein is the sole actopaxin/alpha-parvin ortholog in *C. elegans* and localizes to the main attachment complexes in body wall muscle, the M-lines and dense bodies [20] and WAH-1, is a mitochondrial enzyme [35]. The results are shown in Figure 1B. In both cases, the protein localization that we observed using the gateway method was identical to the previously published data. In addition, we verified the localization of several Gateway constructs by cloning and tagging the corresponding gene via conventional methods using genomic DNA instead of cDNA, and using endogenous promoters instead of our muscle specific promoter. We were able to confirm the same sub-cellular localization using both methods (three examples are shown in Figure 3). However, detection of GFP fluorescence in the body wall muscle cells was frequently covered by strong fluorescence in other tissues (e.g. hypodermis or gut) when using non-muscle specific endogenous promoters (data not shown).

### Sub-cellular localization of proteins within the *C. elegans* muscle cell

We generated transgenic strains carrying expression clones for 307 genes and were able to identify the sub-cellular localization pattern of 231 different GFP-tagged proteins (including the two controls, T21D12.4/*pat-6* and Y56A3A.32/*wah-1*). We did not detect any GFP expression in 76 transgenic strains even though they carried an expression clone, as demonstrated by PCR analysis. Additional 5′ sequencing of these 76 non-expressing clones was done to check for errors in their DNA sequence. Surprisingly, 15 clones had completely wild type sequence and yet no GFP expression was observed (see Table S1 for the gene names of these clones). More typically, clones not resulting in detectable GFP expression had minor sequence errors including amino acid substitutions. The number of defective Gateway clones that we detected here (61 out of 307; ∼20%) should serve as a caution to others who wish to use this particular library.

The main focus of this study was to identify new protein components of muscle specific structures; specifically the myofilament lattice, dense bodies and M-lines. The body wall musculature is the largest tissue in *C. elegans*. An adult hermaphrodite has 95 body wall muscle cells arranged into four quadrants each lying underneath a thin layer of hypodermis adjacent to the cuticle. The myofilament lattices in the body wall muscle cells contain overlapping thick and thin filaments that are connected to the cell membrane by attachment structures called dense bodies and M-lines (reviewed in [9]). In this study we have identified 83 GFP-tagged proteins that localize to one or more of these muscle specific structures. Most of the remaining proteins are located in structures common to all cell types such as the cell membrane, nucleus, cytoplasm, endoplasmic reticulum (ER) and mitochondria. There is also a group of 30 proteins whose localization we could not determine with any degree of certainty. In most cases protein localization is not exclusive to a particular sub-cellular compartment or structure. Finally, two GFP-tagged proteins were not expressed in body wall muscle cells, but instead were found in the pharynx (T01H3.2) or in a few neurons in the head (Y5F2A.2/*ttr-17*). The reason for this non-muscle expression is unclear. The Y5F2A.2 clone has been completely sequenced and no errors were detected. The protein does contain an N-terminal signal sequence so it may be made in the muscle cells and then exported out of the cells. Both ends of the T01H3.2 clone have been sequenced and no errors were found; however, the entire cDNA sequence has not been determined because of the large size of the clone. There may be additional transcriptional regulators within the coding sequence of either gene that have not been identified. We have grouped the muscle expressing GFP-tagged proteins exhibiting similar localization patterns into one of the 14 broad categories described in Table 1. Table S2 lists the proteins assigned to each category and any available data about their known or predicted functions. A list of the proteins in each category is available in Table 2.

**Table 1:** The categories of GFP-tagged protein localization identified in this study.

Category	Localization Pattern	Proteins in Category
1	Myofilaments (+/−Dense bodies)	10
2	Dense bodies, M-lines, Attachment sites	2
3	Dense bodies, Attachment sites	5
4	Dense bodies, Cytoplasm (+/−M-lines, Nucleus)	33
5	Sarcoplasmic reticulum-like	19
6	Dense bodies, Thick filaments and/or M-lines, ER/SR	14
7	Cell membrane (+/−Muscle arms)	30
8	Nucleolus	8
9	Nucleus only	17
10	Nucleus, Cytoplasm or Other	20
11	Mitochondria	25
12	Endoplasmic reticulum (+/−Other)	23
13	Other Cytoplasmic or Cytosol	17
14	Unique undetermined structures	4
15	Non Body wall muscle	2

**Table 2:** Proteins with sub-cellular localization listed by category.

Category: ORF/gene Names
**1:** B0303.2, C04F12.8, C08D8.2§, F09F7.2, F15G9.1, F28H1.2§, R31.2, T20B3.2§, Y17G7B.7*, ZC395.10
**2:** F32A7.3, M01E11.7§
**3:** C52B11.2*, F42H10.3§, M03A8.4, Y71H2AM.15§, ZK353.7*
**4:** C16C10.11, C17G1.7*, C40H1.6§, C47B2.2, C55A6.10, D1007.4*, D2063.1, D2063.3, F22B5.10*, F22F7.7, F25H2.4, F25H2.12, F26A3.4, F46F6.2, F52F12.3, F56B6.4, K02C4.4, K04B12.3, M79.2/3, T01G9.2, T04C9.4, T06D10.1, T22A3.2, W03C9.2, W05G11.6, W06D4.1*, Y37D8A.1§, Y39A1C.1, Y43F8B.2, Y45G5AM.6, Y48G10A.3, ZK637.2§, ZK643.1
**5:** B0412.3*, C29F5.1, C48D5.2, F38B7.1, F44A2.5, F57C2.5, K01A2.3, K07F5.15, K08C7.6, R151.10, T03G6.3, T26E3.2, W01A11.2, Y37D8A.10, Y38F1A.9, Y54E5A.5, Y57G7A.10§, Y106G6A.1§, ZK54.1§
**6:** C04G6.4, C53B4.7* §, D2013.9*, F55C10.1§, H20J04.5, K08E3.5, M02D8.1, R102.5, T05D4.1§, T12D8.8, T22A3.4, W03F9.1, Y57G11C.3, ZK593.1* §
**7:** C05D9.3* §, C05G5.1, C24A3.2, C25D7.8, C29F5.7*, C40C9.5, D1037.4*, F14B6.2, F21C3.1, F25B3.1*, F29B9.8, F52H3.7§, K04G2.9, K07G5.1*, M02B1.3, R07E5.7, R10E4.9, T10C6.6, T27A1.4, W06A7.2, Y9C2UA.1, Y15E3A.4, Y48C3A.16, Y55F3C.3, Y57A10A.16, Y71F9B.3, ZK563.4*, ZK637.3, ZK892.1, ZK1321.2
**8:** K01G5.8, W04C9.4*, W09C5.1§, Y39B6A.33, Y40B1B.7§, Y52B11A.9, Y54G11A.11, ZK265.6
**9:** B0024.10*, C26E6.2, D2030.3*, F13B12.1, F25B3.6*, F25B5.7§, F44G4.4, F53E2.1, K01G5.1, K10C3.6, R07B7.3, T01D1.2, T10C6.5§, T11G6.8§, W05H7.4, ZK1128.5§, ZK1236.7* §
**10:** C28H8.12, C32D5.9§, C47E12.5, C53B4.3*, F01F1.5, F08B12.4, F22D6.2, F25H8.2, K01H12.1, T01C3.2, T01G9.6* §, T20D3.8, T28B4.3*, W03A5.7, Y43F4B.5, Y73B6BL.21§, ZC504.5, ZK856.9, ZK856.11, ZK1098.1§
**11:** B0035.15, C33H5.19, D1007.14, D2030.5, F13D12.4, F20D1.10, F20H11.3*, F21C10.10, F45G2.4, F53F10.1, F54C8.7, F57B10.14, K02A4.1§, K04D7.3, R07H5.3§, R119.3*, T05G5.5§, T09A5.5*, T10B11.6*, T15B12.1§, Y43E12A.2 Y53G8AR.8§, Y66H1A.3, Y67H2A.5, ZC97.1
**12:** C05E4.3§, C35E7.8, C47D12.2, D2024.5, F01G4.5, F02E9.1, F25D7.1§, F25H2.11§, F33A8.3§, F36F2.1, F48E3.8, F52A8.4*, M03F4.7, M05D6.5, M176.4, R04F11.5, T10B10.4, T22C1.6, W04A8.4, W08E3.3, Y105E8A.3, Y113G7B.17*, ZK1127.10§
**13:** B0001.6, C04F12.3, C08F8.1, C49H3.6, F02A9.4, F37H8.5§, F42G9.9, F46F11.1*, F47F2.1*, K07H8.1*, M01E5.4, M18.3, R151.4, T06A10.3*, T20D3.6, Y46G5A.12§, Y54E10BR.4*
**14:** D2092.4, F42C5.9*, K06A4.3§, R11G1.6
**15:** T01H3.2, Y5F2A.2

*Indicates ORF with RNAi phenotype from study by [26].

§ Indicates fly ortholog with RNAi phenotype from study by [46].

**Figure 4:**
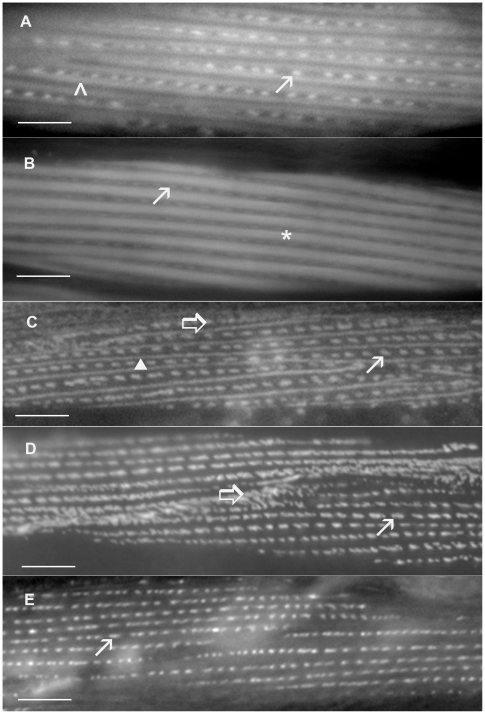
Sub-cellular localization patterns for some category 1, 2, 3 and 4 GFP-tagged proteins. Panels A and B show single symmetrical, spindle–shaped body wall muscle cells exhibiting GFP-tagged protein localization. Panels C, D and E show parts of two adjacent body wall muscle cells exhibiting GFP-tagged protein localization. The category 1 proteins, F15G9.1 (A) and B0303.2 (B), are localized to the myofilament lattice and the dense bodies. The category 2 protein, M01E11.7 (C), is found in the dense bodies, M-lines and cell attachment sites. The category 3 protein, M03A8.4 (D), is a component of the dense bodies and cell attachment sites and the category 4 protein, T04C9.4 (E), is localized to the dense bodies and cytoplasm. The thin arrows point to dense bodies, the thick arrows to the cell attachment sites and the closed arrow head points to the M-line. The asterisk indicates thick filaments and the open arrowhead indicates thin filaments. Bars represent 10 µm.

The GFP-tagged proteins in category 1 all localize to either the thick or thin filaments of the myofilament lattice, and in a few cases, to the dense bodies as well. Three of the proteins in this group are known components of muscle filaments, myosin light chain (F09F7.2/MLC-3), tropomodulin (C08D8.2/TMD-2) and troponin I (T20B3.2/TNI-3; [36]) and therefore, localize as expected. The sub-cellular localization of both the C04F12.8 and R31.2 proteins is remarkably similar to that of the thick filament protein F02F7.2/MLC-3 (myosin light chain). Thus these two proteins are probably associated with the thick filaments. The B0303.2 protein appears to be associated with the thick filaments and dense bodies while the F15G9.1 gene product is associated with the thin filaments and dense bodies (shown in Figure 4A and 4B).

The category 2 pattern is similar to that seen with PAT-3/β-integrin [37], UNC-112 [18], and the many other proteins that form the dense bodies, M-lines and attachment sites between adjacent muscle cells (reviewed in [8]; shown in Figure 4C). The category 3 GFP-tagged proteins localize to dense bodies and cell- cell attachment sites in a pattern similar to the Deb-1/vinculin protein [15] (shown in Figure 4D) while in category 4 proteins localize mainly to dense bodies, although in some cases there appears to be faint, inconsistent M-line expression as well (see Figure 4E).

**Figure 5:**
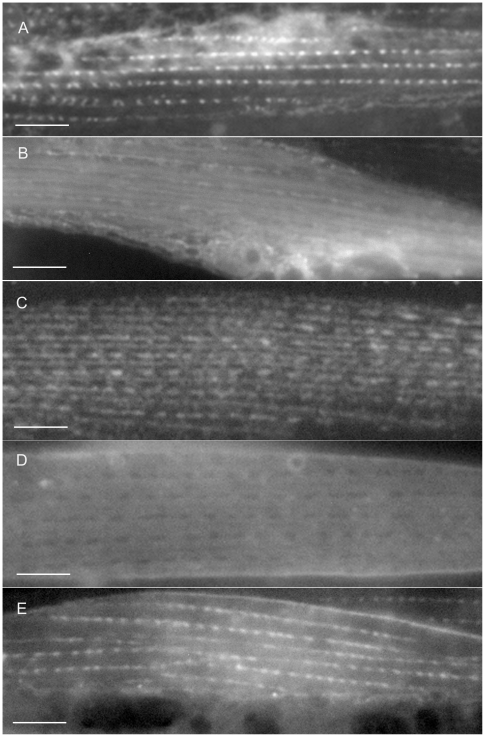
Sub-cellular localization patterns for some category 5, 6 and 7 GFP-tagged proteins. All 5 panels show single symmetrical, spindle–shaped body wall muscle cells exhibiting GFP-tagged protein localization. The category 5 protein, T03G6.3, (A), is most likely a component of the sarcoplasmic reticulum. The category 6 protein, T22A4.3, (B), is found in the dense bodies, thick filaments and/or M-lines and the ER/SR. The category 7 proteins, Y71F9B.3 (C), ZK637.3 (D) and R07E5.7 (E), all localize to the muscle cell membrane in various patterns. Bars represent 10 µm.

The *sca-1* gene in *C. elegans* encodes a component of the sarcoplasmic reticulum (SR), sheet-like membranous sacs which lie against the plasma membrane and extend into the lattice along the sides of the dense bodies [9]. The expression patterns of the GFP-tagged proteins assigned to category 5 are very similar to the SCA-1::GFP pattern described by Zwaal et al. [38]. Thus some or all of the genes in this category may encode components of the SR (see Figure 5A). The GFP-tagged proteins in category 6 are located in several places throughout the cell. They appear to be in the dense bodies, M-line and/or thick filaments, as well as the ER or SR (see Figure 5B). Examples of the various localization patterns from categories 1 through 6 are shown in Figures 4 and 5.

Most of the remaining GFP-tagged proteins locate to sub-cellular compartments that are common to all types of cells. The category 7 GFP-tagged proteins are present throughout the muscle cell membrane including, in some cases, the muscle arms. This is a large and diverse group of proteins and some of the localization patterns that we observed are shown in Figure 5. Several of the proteins in this category appear to be associated with dense body-like structures (for example R07E5.7 in Figure 5E) while others appear to localize everywhere except the dense bodies (for example ZK637.3 in Figure 5D). The GFP-tagged Y71F9B.3 gene product localizes as dashes throughout the cell membrane and may also be present in the myofilament lattice (shown in Figure 5C). This protein is similar to YOP-1, a yeast protein involved in membrane trafficking.

**Figure 6:**
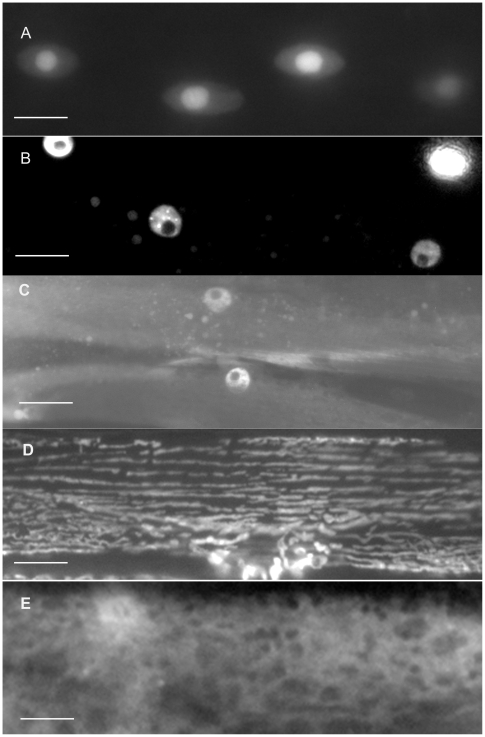
Sub-cellular localization patterns for some category 8, 9, 10, 11 and 12 GFP-tagged proteins. The category 8 protein, W04C9.4 (A), localizes to the nucleolus, and the category 9 protein, K10C3.6, (B), localizes exclusively to the nucleus. The category 10 protein, F22D6.2, (C), localizes to the nucleus and the cytoplasm. Four muscle cells are shown in panels A and B and two muscle cells are shown in panel C. The category 11 protein, T10B11.6, (D), localizes to the mitochondria. The category 12 protein, C05E4.3, (E), localizes to the endoplasmic reticulum. Panels D and E each show one spindle-shaped muscle cell. Bars in A and B represent 20 *u*m. Bars in C, D and E represent 10 µm.

**Figure 7:**
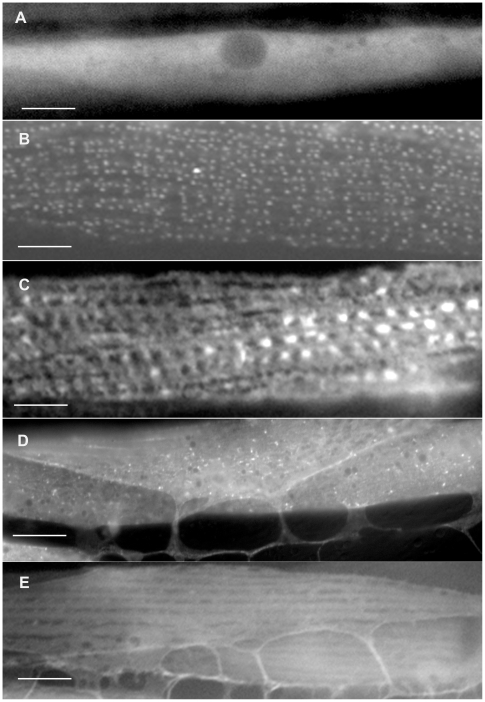
Sub-cellular localization patterns for some category 13 and 14 GFP-tagged proteins. Panels A, B and C show single symmetrical, spindle–shaped body wall muscle cells exhibiting GFP-tagged protein localization. Panels D and E show two adjacent body wall muscle cells exhibiting GFP-tagged protein localization. The category 13 protein, F47F2.1, (A), localizes to the cytoplasm or cytosol. The four category 14 proteins exhibit unique and unusual localization patterns. The D2092.4 protein (B) localizes to organized dots associated with the myofilament lattice. The R11G1.6 protein (C) localizes to ridges and dots in the muscle cell membrane. The K06A4.3 protein (D) appears to localize to the actin cytoskeleton and dense bodies. The F42C5.9 protein (E) is present in the cell membrane, as well as filament-like structures and dense body-like structures. Bars represent 10 µm.

The proteins in category 8 are exclusively expressed in the nucleolus whereas the proteins in category 9 are exclusively expressed in the nucleus. The category 10 GFP pattern is also nuclear but not exclusively, as proteins assigned to this group are also found in the cytoplasm, ER or mitochondria. Examples of the various expression patterns from categories 8, 9 and 10 are shown in Figure 6 (A–C). The GFP-tagged proteins assigned to category 11 are located in the mitochondria (see Figure 6D). Schaheen et al. [39] have shown that F25D7.1/CUP-2 localizes to the ER in *C. elegans* ceolomocytes. We have obtained expression of this protein in muscle cells and it also appears to localize to the ER as well as to dashes in the membrane. GFP-tagged proteins with localization patterns similar to that of F25D7.1 have been assigned to the ER (category 12; shown in Figure 6E). All but three of the remaining GFP-tagged proteins localize either to other undetermined cytoplasmic structures or the cytosol (category 13). An example of one of the expression patterns from category 13 is shown in Figure 7A.

The four proteins included in category 14 exhibit unique expression patterns and it is not clear at this time precisely where they are located (see Figure 7). The F42C5.9 gene product is an actin related protein that is present in the cell membrane, in filament-like structures and in dense body-like structures (Figure 7E). K06A4.3/gelsolin is an actin regulatory protein that appears to be associated with the actin cytoskeleton as well as dense body-like structures (Figure 7D). The D2092.4 protein is a thioredoxin/protein disulfide isomerase that localizes as organized dots associated with the myofilaments (Figure 7B) whereas the R11G1.6 protein appears to localize to ridges and dots in the muscle cell membrane (Figure 7C).

The most common pattern that we observed was dense body-like (category 4). A total of 33 GFP-tagged proteins appear to be localized to the dense bodies (+/−M-lines) including 16 uncharacterized proteins and 16 predicted enzymes (Table S2). Thirty of the GFP-tagged proteins localize to the muscle cell membrane in a number of different patterns including, in some cases, the muscle arms (category 7). Twelve of these are predicted membrane proteins based on the analysis of their amino acid sequence (data from www.wormbase.org, release WS215 [40]). Seventeen GFP-tagged proteins localized exclusively to the nucleus (category 9) and another 19 localize to the nucleus and either the cytoplasm, ER or mitochondria (category 10). There are eight GFP-tagged proteins in the nucleolus (category 8), five of which are predicted ribosomal proteins and one is a transcription elongation factor.

**Figure 8:**
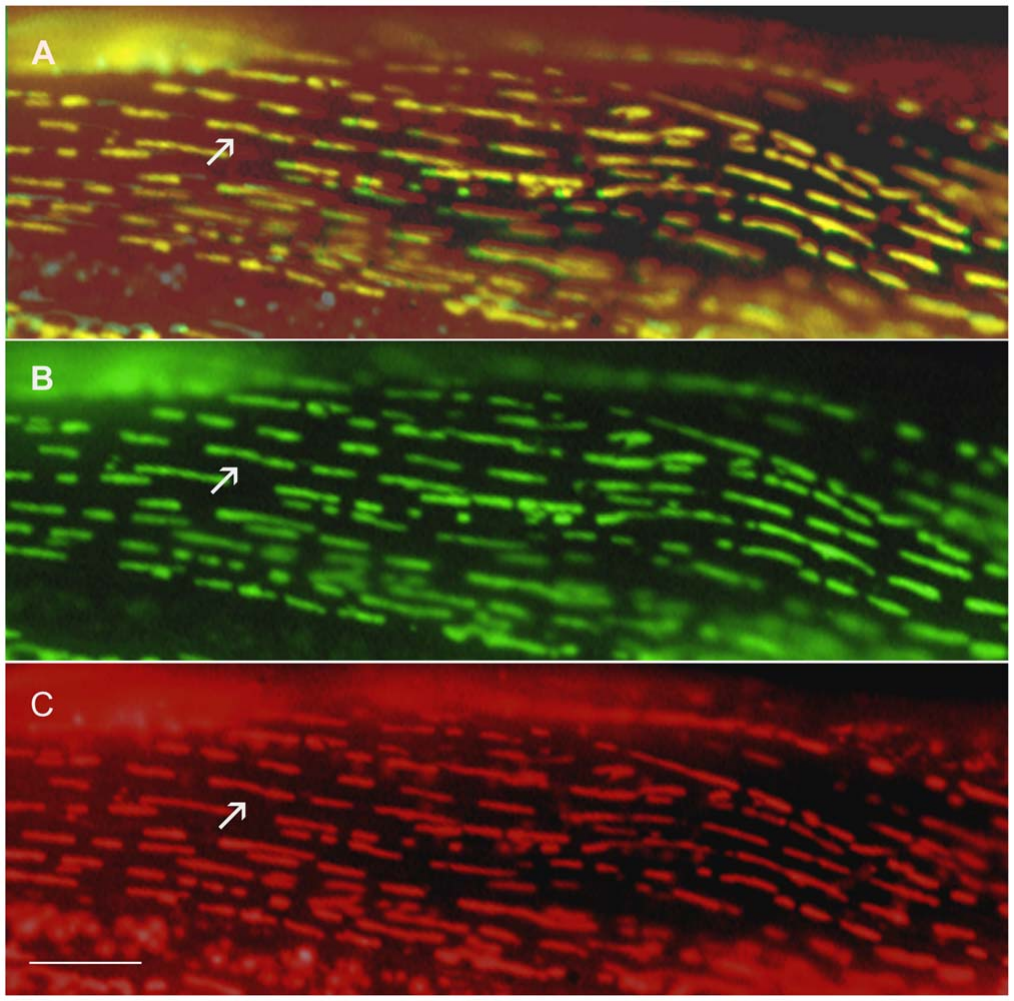
The GFP-tagged F21C10.10 protein co-localizes with the MitoTracker dye in mitochondria. Panel A shows the co-localization of the F21C10.10 GFP-tagged protein (green) with the MitoTracker dye (red) in mitochondria in the same body wall muscle cell. Panel B shows the GFP localization pattern of F21C10.10 (shown in green) in a body wall muscle cell in an animal from the DM7305 strain. Panel C shows the mitochondria staining with the MitoTracker dye (shown in red) in the same body wall muscle cell. The arrow points to a single mitochondrion. Bars represent 10 µm.

Twenty-five proteins have been assigned to the mitochondria (category 11), the third most common identified pattern observed in this study. Less than half (10/25) of the category 11 proteins are orthologs of proteins known to be located in the mitochondria in other organisms. We have confirmed the localization of 17 proteins by co-staining animals with the mitochondria specific stain, Mitotracker [41]. In each of these cases, the staining pattern observed with Mitotracker was identical to the pattern exhibited by the GFP-tagged proteins indicating mitochondrial expression (Table 3). The Mitotracker staining result for the F21C10.10 protein is shown in Figure 8. We also determined whether any of the category 11 proteins contain the Mitochondrial Targeting Sequences (MTS; [42]) found in some proteins that localize to the mitochondria. Twenty-three of the proteins identified in this study were analyzed using the MitoProt [43] and TargetP 1.1 [44], [45] MTS prediction programs, and they identified 14 and 6 MTS-containing proteins, respectively (Table 3). Since all six of the MTS signals identified by TargetP were also identified by MitoProt there are at least 9 proteins without an apparent MTS in our set of mitochondrial proteins.

**Table 3:** Analysis of Mitochondrial proteins.

ORF Name	Localization confirmed by MitoTracker	MTS predicted by MitoProt	MTS predicted by TargetP
B0035.15	Yes	Yes	No
C33H5.19	Yes	Yes	Yes
D1007.14	Yes	No	No
D2030.5*	Yes	Yes	Yes
F13D12.4*	Yes	Yes	Yes
F20D1.10*	Yes	Yes	No
F20H11.3*	Not Done	Yes	No
F21C10.10	Yes	No	No
F53F10.1	Not Done	No	No
F54C8.7	Yes	No	No
F57B10.4	Yes	No	No
K02A4.1*	Not Done	Yes	Yes
K04D7.3	Yes	Yes	No
R07H5.3*	Yes	No	No
R119.3*	Yes	Yes	No
T09A5.5	Yes	No	No
T10B11.6	Yes	Yes	Yes
T15B12.1	Yes	No	No
Y43E12A.2	Not Done	No	No
Y53G8AR.8*	Yes	Yes	Yes
Y66H1A.3*	Not Done	Yes	No
Y67H2A.5	Yes	Yes	Yes
ZC97.1*	Not Done	No	No

*Indicates ORF with known mitochondrial ortholog.

Another common pattern observed in this study is the endoplasmic reticulum (ER). Three categories include this putative ER expression either on its own (category 12; 23 proteins), with the SR (category 5; 19 proteins) or with the dense bodies and myofilaments/M-lines (category 6; 14 proteins). The F25D7.1 [39] and F02E9.1 (www.wormbase.org, release WS215; [40]) gene products that we have placed in category 12 are the only predicted ER proteins among those characterized in this study. Twenty-seven GFP-tagged proteins localize to undetermined cytoplasmic structures (category 13). Two of these proteins are predicted microtubule associated proteins and another is an intra-cellular GTP-binding protein. Also in this category is the F37H8.5 gene product. This protein is predicted to be a lysosomal thiol reductase and the localization pattern that we observed is consistent with lysosomal localization and function. None of the other proteins in this study exhibit the F37H8.5::GFP localization pattern.

The GFP-tagged proteins in categories 1, 2 and 3 exhibit localization patterns similar to approximately 20 known proteins of body wall muscle sarcomeres. Most of these proteins are essential for muscle development and were identified through genetic screens for mutants exhibiting arrested, or abnormal muscle development (reviewed by [8]–[10]). In this study we have identified an additional 14 GFP-tagged proteins that exhibit one of these three expression patterns. Five proteins localize to the myofilaments, five proteins localize to the dense bodies and the cell-cell attachment sites between adjacent muscle cells and two proteins localize to the dense bodies, M-lines and cell-cell attachment sites. The remaining two GFP-tagged proteins, encoded by B0303.2 and F15G9.5, localize either to the thick filaments plus the dense bodies or to the thin filaments plus the dense bodies, patterns not previously observed (shown in Figure 4A and 4B). The final four proteins, D2092.4, F42C5.9, K04A6.3, and R11G1.6, were assigned to a separate category [14] based on their unique and unusual localization patterns (see Figure 7B–7E). In these cases the specific sub-cellular structures identified by the GFP-tagged proteins are unknown.

**Figure 9:**
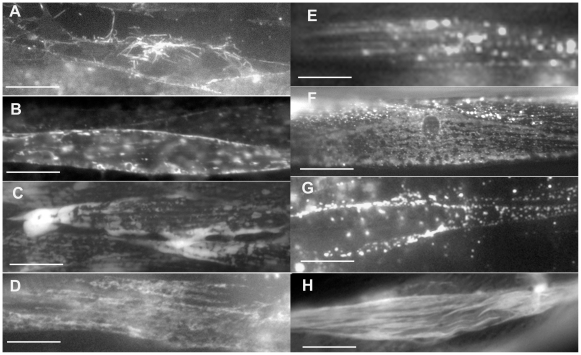
GFP-tagged proteins with apparently disorganized localization. Some of the GFP-tagged proteins with apparently disorganized localization including the myofilaments (H) and muscle cell membrane (A and B). (A) F29B9.8; (B) F52H3.7; (C) F56B6.4; (D) F38B7.1; (E) F02A9.4; (F) F33A8.3; (G) F37H8.5; (H) ZC395.10. Several body wall muscle cells are shown in panels A, C and D and single muscle cells are shown in the remaining panels. Bars represent 10 µm.

There are at least two caveats we must consider in this type of analysis. The first being the presence of the GFP tag at the carboxy-terminus of the proteins characterized in this study, and the second being protein over expression from transgenic arrays. Either or both of these factors may cause issues with protein localization as well as function. Some of the localization patterns observed in this study are quite disorganized indicating that the presence or perhaps over expression of the GFP-tagged protein may be disruptive. Some examples of this are shown in Figure 9. The two most pronounced are ZC395.10 (hsp-90 co-chaperone) and F29B9.8 (predicted membrane protein). The myofilaments in animals expressing ZC395.10::GFP appear wavy and disorganized when viewed with fluorescence microscopy (Figure 9H), and transgenic animals expressing this protein exhibit uncoordinated movement. The F29B9.8::GFP protein appears as disorganized clumps (Figure 9A) and again transgenic animals expressing this protein exhibit a mutant phenotype. In addition, the myofilaments in these transgenic animals appear disorganized when viewed with polarized light microscopy (data not shown). Other proteins with very disorganized GFP expression include F02A9.4 (COA carboxylase) (Figure 9E), F33A8.3 (RNA-binding protein) (Figure 9F), F38B7.1 (Zn-finger protein) (Figure 9D), F56B6.4/*uvt-5* (glycosyl transferase) (Figure 9C) and F52H3.7/*lec-2* (tandem repeat type lectin) (Figure 9B).

**Figure 10:**
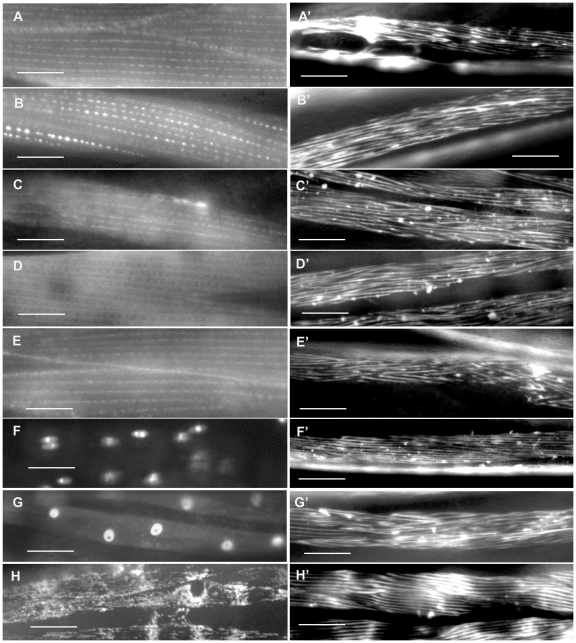
Disorganized body wall muscle phenotypes after RNA interference. Some examples of the disorganized MYO-3::GFP phenotypes that were observed in the RNAi study by Meissner et al [26] are shown (on the right) with the corresponding ORF::GFP sub-cellular localization pattern from this study (on the left). Examples of wild type myofilament structure are shown in Figure 2A and 2B. (A, A') C52B11.2, category 3; (B, B') W06D4.1, category 4; (C, C') B0412.3, category 5; (D, D') ZK593.1, category 6; (E, E') D1037.4, category 7; (F, F') W04C9.4, category 8; (G, G') B0024.10, category 9; (H, H') T10B11.6, category 11. Several body wall muscle cells are shown in most panels. Bars in A through E represent 10 µm. Bars in F through H and A' through H' represent 20 µm.

In an earlier study we identified 108 new genes that affect muscle structure after knockdown using RNAi [26]. We were able to obtain protein sub-cellular localization data for 37 of the muscle affecting genes identified in that study (Table 2; Figure 10). One of these genes is D2013.9 or *ttll-12*, which encodes a member of the tubulin-tyrosine ligase family of proteins. The loss or reduction of the D2013.9 gene product by RNAi knockdown results in a very severe disruption of the myofilament lattice. We find this protein in the dense bodies, thick filaments and/or M-line and in the SR/ER. Another gene, T28B4.3/*ttr-6*, encodes a member of the transthyretin-like family of uncharacterized proteins. The loss of this protein by RNA interference results in arrest at the two-fold stage of embryogenesis (i.e. the Pat phenotype) and the data obtained here show the TTR-6 protein to be present in the nucleus, cytoplasm and possibly the M-line. Some examples of the RNAi phenotypes that were observed by Meissner et al [26] are shown with the corresponding protein sub-cellular localization pattern in Figure 10.

A similar, albeit larger study using RNAi in *D. melanogaster* identified over 1700 genes required for wild type muscle structure in flies [46]. Just over half (116/227) of the genes in our study have an ortholog that was assayed for an RNAi phenotype in that study. Although the majority of the assays resulted in no phenotype there were 42 that did have an effect on muscle structure. The *C. elegans* orthologs of the 42 muscle-affecting genes from the fly are indicated in Table 2. Five of these 42 worm genes were among the 108 muscle-affecting genes identified by Meissner et al [26] while the other 37 either were not tested or did not have a phenotype in that study.

## Discussion

In this study we have provided sub-cellular localization data for 227 proteins in the body wall muscle cells of the nematode *C. elegans*. The sub-cellular localization of a small percentage of these proteins was predicted or known from other recent studies; however, for the majority of the proteins the only available sub-cellular localization information is provided here. We have grouped proteins with similar expression patterns into one of 14 different categories. Many sub-cellular compartments are well documented in the nematode and we feel confident in assigning proteins to these specific regions. These include the nucleus, nucleolus, mitochondria and many of the structures that make up the muscle sarcomere. In other cases our assignments should be viewed as tentative. In particular, there is surprisingly little data available describing Golgi, lysosomal or peroxisomal expression in *C. elegans*. Our analysis in this current study should be very useful in characterizing these structures in nematode muscle cells. New antibodies that identify components of some of these structures have recently become available and they can be used in future for co-localization experiments [47]. Finally, although some of the expression patterns that we observe are similar they are not identical, and thus some of the proteins in a particular group may localize to different structures, or perhaps different parts of the same structure. Additional work will be required in the future to identify the precise location and function of some of the more problematic proteins.

For this project we primarily utilized ‘Gateway cloning’ rather than the more time consuming cut and paste cloning method. The availability of a *C. elegans* ORFeome library made this large-scale project possible and required only the construction of a destination vector to allow expression within the body wall muscle cells. Although the ORFeome clones offer ease of use they do have limitations. Primarily, not all of the ORF's in the genome are represented in this library. This occurs for various reasons, but is mainly due to the difficulty of obtaining full-length cDNA clones for very large genes. Another approach to obtain GFP-tagged protein expression is recombineering, a protocol adapted for *C. elegans* by Dolphin and Hope [48] and Sarov et al. [49]. The advantage of recombineering is that the GFP encoded sequences are inserted into large (30 to 40 kb) genomic DNA fragments cloned into fosmid vectors (data available at http://elegans.bcgsc.bc.ca/perl/fosmid/CloneSearch). We should be able to use this protocol in future to characterize muscle genes not present in the ORFeome library.

In this study we were successful in obtaining sub-cellular localization data for 231 proteins (including controls and non-muscle expression; Table S1), about 59% of the 390 proteins in our original list. We incurred losses at two steps in our protocol. First, about 21% of the expression clones that we made using donor clones from the *C. elegans* ORFeome library were found to be defective after our first sequencing step. None of those clones were used for microinjections. Second, another 19.8% of the expression clones failed to produce any observable GFP-tagged protein expression in transgenic animals. In our hands, using the Gateway method of cloning and the commercially available nematode ORFeome library we obtained a 59% success rate. The amount of time and effort required to obtain good clones for microinjections using our protocol is minimal compared with that required to obtain and analyze transgenic animals, thus a 21% loss at the first stage is not a major setback. When we eliminate the faulty clones from our calculations we find that 75% of the clones that were used for microinjections produced reliable sub-cellular localization data. We believe this reasonably high success rate justifies the effort involved.

Ghosh and Hope [50] recently published a similar, although much smaller, study of sub-cellular protein localization in muscle cells. That study also utilized the Gateway method of cloning and the commercially available ORFeome library. However, a major difference was that instead of using a single muscle promoter like we did in our study they used endogenous promoters. Ghosh and Hope [50] constructed and injected 62 clones encoding full length GFP fusion proteins and obtained expression patterns for 37 of them (∼60%). Just under half of their transgenic lines (16/37) exhibited expression in muscle cells, and of these only three, C46G7.2, K04A8.6 and T03G6.3, were located in the sarcomere and characterized further. Two of these genes were in our list and we obtained transgenic lines for both of them. Unfortunately, none of our four lines carrying K04A8.6 showed any GFP expression even though the clone was shown to be present by PCR analysis. Transgenic lines carrying the T03G6.3 clone exhibited good expression (see Figure 5A) and this gene was assigned to category 5 as a possible component of the sarcoplasmic reticulum. Our sub-cellular expression data for T03G6.3 is very similar to the data obtained in the Ghosh and Hope [50] study for their ORFeome (cDNA) clone as well as a full-length genomic clone.

At least 80 of the proteins characterized here are new components of known muscle specific structures, including the myofilaments, dense bodies, M-lines, cell-cell attachment structures and the sarcoplasmic reticulum. This more than doubles the number of proteins previously shown to localize to these structures and identifies many interesting genes for further analysis. Of particular interest are the many conserved proteins of unknown function. Confirming that we have correctly identified the sub-cellular localization of these proteins to known muscle specific structures should be straightforward since there are a number of antibodies available for co-localization experiments. However, determining whether the location of the protein seen here actually reflects the location of the protein *in vivo* is more challenging. After all, the GFP molecule is 200 amino acids in size and could interfere with protein localization as well as protein function. Remarkably it has been our experience and the experience of many others that GFP interference with a protein occurs in only a minority of cases. In future we hope to confirm the sub-cellular localization identified in this study by making antibodies to the protein in question and/or by demonstrating rescue of a mutant phenotype by the GFP-tagged fusion protein.

A significant number of the genes in this study have deletion alleles isolated by the *C. elegans* gene knockout consortium (reviewed in [51]), thus allowing us to determine what effect the absence of a particular protein has on muscle structure. A case in point is B0303.2 a gene encoding an N-methytransferase that localizes to the myofilaments and dense bodies (category 1). In the absence of this protein the myofilament lattice is mildly disorganized but the animals develop normally and move well (T. M. Rogalski and D.G. Moerman, unpublished results). RNA interference is a commonly used method to reduce or eliminate gene expression. We have identified the sub-cellular localization of 37 proteins shown to be required for wild type muscle structure in *C. elegans* by RNA interference [26], as well as another 37 proteins that have orthologs shown to be required for wild type muscle structure in flies, again by RNA interference [46]. Knowing the sub-cellular localization of a particular protein and the type of muscle disorganization that occurs in the absence of that protein is a major step in determining its role in muscle development. Presumably the majority of fly proteins identified by Schnorrer et al. [46] will have the same sub-cellular localization in muscle cells as their worm orthologs.

The study described here is, to our knowledge, the largest sub-cellular protein localization study in *C. elegans* and the first to specifically target muscle cells in any organism. Similar large-scale protein localization studies have been carried out in several organism including Yeast [28], Drosophila [52], plants [53]–[55] and cultured mammalian cells [56], [57]. The largest study is in *S. cerevisae* where Huh et al. [28] were able to obtain GFP-tagged protein localization data for over 4,100 proteins representing 75% of the yeast proteome. About 40% (91/227) of the ORFs in our study have some homology to yeast proteins and, in some cases, have similar sub-cellular localization.

A major contribution of our work is the identification of several new sub-cellular localization patterns in body wall muscle cells. The D2092.4::GFP, F42C5.9::GFP, K06A4.3::GFP and R11G1.6::GFP proteins in particular exhibit unique and unusual sub-cellular localization patterns. The body wall muscle cells are relatively large and abundant compared to most of the other cell or tissues types in *C. elegans*, characteristics that make them ideal for studying sub-cellular protein localization. The myofilament lattice is attached to the cell membrane adjacent to the hypodermis and cuticle, and the rest of the muscle cell consists of the nucleus, cytoplasm and various organelles. Ninety-seven GFP-tagged proteins from this study localize only to structures located in the cytoplasm including the nucleus, and at least 30 proteins appear to be associated with the plasma membrane. Although these proteins are all expressed in body wall muscle (based on SAGE and/or microarray data) it is unlikely that the majority of them are specific to this tissue. The potential of this system extends beyond muscle cells and could easily be used for studying the sub-cellular localization of proteins specific to other types of cells. For example, since the small size of neurons makes it very difficult to determine sub-cellular protein localization, expressing neuron specific proteins in muscle cells could determine their sub-cellular location and thus help to elucidate their function.

## Materials and Methods

### Molecular constructs and transgenic strains

The Gateway destination vector (pDM#834) was constructed by the following method. Firstly, an 1,878 bp promoter region upstream of T05G5.1 was amplified from wild type (N2) genomic DNA using primers T05G5.1-Fo-Hind, TACTT*AAG-CTT*TTCCTATCTCCG-3 and T05G5.1-Re-XmaI, TCC*CCCGGG*GCCTGAAG-ATAAGTGTGAA, and then inserted between the *HindIII* and *XmaI* sites of the GFP-encoding vector pPD95.75 (Fire LabVector Kit available at http://www.addgene.org/pgvec1?f=c&cmd=showcol&colid=1) to generate pDM#823. A second PCR fragment containing the attR sites and the ccdB gene from the pDEST24 destination vector (nucleotides 70–1777; Invitrogen) was amplified and cloned into p#DM823 between the MscI and KpnI cloning sites to generate pDM#834. This plasmid was transformed into the *E. coli* strain DB3.1 (Invitrogen), which is tolerant for the *ccdB* selectable marker gene. Entry clones were obtained from the ORFeome project (Open Biosystems) and cloned into the destination vector pDM#834 using the gateway strategy with LR clonase (Invitrogen) to make the pT05G5.1::ORF::GFP expression clones. The sequences of the primers used to amplify the required fragment from pDEST24 are: CA*GGCGCC*ACAAGTTTGTACAAAAAAGCTGAAC and GG*GGTACC*CCCCTCACCACTTTGTACAAGAAAGCTG. The pDM#834 destination vector will be available through Addgene (http://www.addgene.org/).

Genomic clones encoding GFP-tagged fusion proteins were constructed for three genes using the following method. The entire genomic coding sequences of these genes, including their endogenous promoters, were amplified from genomic N2 DNA by PCR and then cloned in frame into the pPD95.75 GFP expression vector. The sequences of the primer sets that were used to construct the genomic clones are as follows. For the D1007.14 gene: D1007.14_Fo_HindIII: CCCAAGCTTGCTCAAGAAAGTTTTGCACACG, and D1007.14_Re_XmaI: CATCCCGGGGGGACTTTCCAGTAGTAGGAC; for the T05D4.1 gene: T05D4.1_Fo_HindIII: CCAAAAGCTTCTAAAACTTGC, and T05D4.1_Re_XmaI: CATCCCGGGGAGAATGATTGGCGACGAAGAGG; for the D2030.5 gene: D2030.5_Fo_PstI: AACTGCAGCGACATCTATTCAAGCAGTGGC and D2030.5_Re_XmaI: CATCCCGGGGCTGCTCGAGTTCAATAAGTAC. All of the clones generated in this study are listed in Table S1.

Microinjections were performed according to Mello and Fire [34]. In the majority of cases, the Gateway plasmid DNAs were co-injected with the *pha-1* rescuing plasmid, pBX [58], and the pRF4 [*rol-6*(*su1006*)] plasmid into the gonad syncytium of *pha-1*(*e2123*ts)III hermaphrodites. Injected animals were incubated at 25°C to select for rescue of the Pha-1 lethal arrest phenotype by the pBX plasmid DNA [58]. Alternatively, some plasmid DNAs were co-injected into N2 hermaphrodites with just the pRF4 [*rol-6*(*su1006*)] plasmid and F1 Rol animals were selected from among the progeny of the injected animals. All of the transgenic *C. elegans* strains constructed in this study are listed in Table S1. These strains will be available through the Caenorhabditis Genetics Center (http://www.cbs.umn.edu/CGC/).

### In vivo analysis and imaging of GFP-expressing animals

Fluorescent imaging of GFP expression and sub-cellular localization was done with either a Zeiss Axioplan or a Zeiss Axiophot microscope. Images were captured on a digital camera using QICAM, (QImaging, www.qimaging.com) and QCapture software.

### Mitochondria Staining with MitoTracker

The mitochondria in live GFP-expressing animals were stained with the MitoTracker dye (Invitrogen) using the following protocol. Worms were washed off of plates, gently centrifuged until a lose pellet formed and then re-suspended in 1 uM MitoTracker dye in M9 buffer. All samples were shielded from light after this step due to the light sensitivity of the dye. Samples were placed on a rotator at room temperature for two hours, and then allowed to recover on plates seeded with E. coli OP50 for at least 1 hour at room temperature before imaging. Imaging of the MitoTracker staining was done with a Zeiss Axioplan fluorescence microscope as described above.

## Supporting Information

Table S1 **Gateway clones and transgenic strains utilized in this study.**
(XLS)

Table S2 **Proteins with sub-cellular localization in C. elegans body wall muscle cells.**
(XLS)
